# Supplementary material for: Pyruvate Dehydrogenase Kinase 1 inhibition mediated oxidative phosphorylation enhancement in cartilage promotes osteoarthritis progression
Source: BMC Musculoskelet Disord. 2023 Jul 20;24:597. doi: 10.1186/s12891-023-06585-6 (PMC10357736; doi:10.1186/s12891-023-06585-6)
Supplement: Supplementary file 1 — Additional file 1. [file 12891_2023_6585_MOESM1_ESM.zip › MF.Gsea.1653627316242/GOMF_ATPASE_COUPLED_CATION_TRANSMEMBRANE_TRANSPORTER_ACTIVITY.html]

Details for gene set GOMF\_ATPASE\_COUPLED\_CATION\_TRANSMEMBRANE\_TRANSPORTER\_ACTIVITY[GSEA]

|  || Dataset | 55457\_collapsed\_to\_symbols.cls.cls #OA\_versus\_normal.cls.cls #OA\_versus\_normal\_repos |
| Phenotype | cls.cls#OA\_versus\_normal\_repos |
| Upregulated in class | OA |
| GeneSet | GOMF\_ATPASE\_COUPLED\_CATION\_TRANSMEMBRANE\_TRANSPORTER\_ACTIVITY |
| Enrichment Score (ES) | 0.4012567 |
| Normalized Enrichment Score (NES) | 1.5149683 |
| Nominal p-value | 0.040302265 |
| FDR q-value | 0.19010596 |
| FWER p-Value | 1.0 |
Table: GSEA Results Summary

  

Fig 1: Enrichment plot: GOMF\_ATPASE\_COUPLED\_CATION\_TRANSMEMBRANE\_TRANSPORTER\_ACTIVITY      
 Profile of the Running ES Score & Positions of GeneSet Members on the Rank Ordered List

  

| SYMBOL | TITLE | RANK IN GENE LIST | RANK METRIC SCORE | RUNNING ES | CORE ENRICHMENT || 1 | ATP6V1C1 | ATPase H+ transporting V1 subunit C1 [Source:HGNC Symbol;Acc:HGNC:856] | 17 | 1.125 | 0.0890 | Yes |
| 2 | ATP6V0D1 | ATPase H+ transporting V0 subunit d1 [Source:HGNC Symbol;Acc:HGNC:13724] | 135 | 0.872 | 0.1500 | Yes |
| 3 | ATP6V1E1 | ATPase H+ transporting V1 subunit E1 [Source:HGNC Symbol;Acc:HGNC:857] | 287 | 0.758 | 0.1992 | Yes |
| 4 | ATP6V1G1 | ATPase H+ transporting V1 subunit G1 [Source:HGNC Symbol;Acc:HGNC:864] | 515 | 0.655 | 0.2342 | Yes |
| 5 | ATP6V0E2 | ATPase H+ transporting V0 subunit e2 [Source:HGNC Symbol;Acc:HGNC:21723] | 532 | 0.647 | 0.2849 | Yes |
| 6 | ATP6V1D | ATPase H+ transporting V1 subunit D [Source:HGNC Symbol;Acc:HGNC:13527] | 750 | 0.581 | 0.3147 | Yes |
| 7 | ATP1B1 | ATPase Na+/K+ transporting subunit beta 1 [Source:HGNC Symbol;Acc:HGNC:804] | 1177 | 0.481 | 0.3202 | Yes |
| 8 | ATP6V0E1 | ATPase H+ transporting V0 subunit e1 [Source:HGNC Symbol;Acc:HGNC:863] | 1207 | 0.474 | 0.3561 | Yes |
| 9 | ATP6V1H | ATPase H+ transporting V1 subunit H [Source:HGNC Symbol;Acc:HGNC:18303] | 1304 | 0.457 | 0.3854 | Yes |
| 10 | ATP12A | ATPase H+/K+ transporting non-gastric alpha2 subunit [Source:HGNC Symbol;Acc:HGNC:13816] | 2330 | 0.323 | 0.3317 | Yes |
| 11 | ATP2A1 | ATPase sarcoplasmic/endoplasmic reticulum Ca2+ transporting 1 [Source:HGNC Symbol;Acc:HGNC:811] | 2781 | 0.272 | 0.3185 | Yes |
| 12 | ATP6V0C | ATPase H+ transporting V0 subunit c [Source:HGNC Symbol;Acc:HGNC:855] | 2790 | 0.271 | 0.3396 | Yes |
| 13 | ABCC8 | ATP binding cassette subfamily C member 8 [Source:HGNC Symbol;Acc:HGNC:59] | 2844 | 0.266 | 0.3569 | Yes |
| 14 | ATP2B2 | ATPase plasma membrane Ca2+ transporting 2 [Source:HGNC Symbol;Acc:HGNC:815] | 3023 | 0.247 | 0.3629 | Yes |
| 15 | ATP5F1B | ATP synthase F1 subunit beta [Source:HGNC Symbol;Acc:HGNC:830] | 3024 | 0.247 | 0.3828 | Yes |
| 16 | ATP4B | ATPase H+/K+ transporting subunit beta [Source:HGNC Symbol;Acc:HGNC:820] | 3160 | 0.234 | 0.3911 | Yes |
| 17 | ATP2B3 | ATPase plasma membrane Ca2+ transporting 3 [Source:HGNC Symbol;Acc:HGNC:816] | 3263 | 0.225 | 0.4013 | Yes |
| 18 | ATP6V1F | ATPase H+ transporting V1 subunit F [Source:HGNC Symbol;Acc:HGNC:16832] | 3833 | 0.173 | 0.3709 | No |
| 19 | ATP2B4 | ATPase plasma membrane Ca2+ transporting 4 [Source:HGNC Symbol;Acc:HGNC:817] | 3998 | 0.156 | 0.3707 | No |
| 20 | ATP7A | ATPase copper transporting alpha [Source:HGNC Symbol;Acc:HGNC:869] | 4814 | 0.086 | 0.3143 | No |
| 21 | ATP1A3 | ATPase Na+/K+ transporting subunit alpha 3 [Source:HGNC Symbol;Acc:HGNC:801] | 5475 | 0.035 | 0.2658 | No |
| 22 | ATP7B | ATPase copper transporting beta [Source:HGNC Symbol;Acc:HGNC:870] | 5751 | 0.012 | 0.2453 | No |
| 23 | ATP8A1 | ATPase phospholipid transporting 8A1 [Source:HGNC Symbol;Acc:HGNC:13531] | 6256 | -0.024 | 0.2081 | No |
| 24 | ATP13A2 | ATPase cation transporting 13A2 [Source:HGNC Symbol;Acc:HGNC:30213] | 6336 | -0.031 | 0.2045 | No |
| 25 | ATP2A3 | ATPase sarcoplasmic/endoplasmic reticulum Ca2+ transporting 3 [Source:HGNC Symbol;Acc:HGNC:813] | 6559 | -0.046 | 0.1909 | No |
| 26 | ATP6V1B2 | ATPase H+ transporting V1 subunit B2 [Source:HGNC Symbol;Acc:HGNC:854] | 7074 | -0.086 | 0.1579 | No |
| 27 | ATP6V1A | ATPase H+ transporting V1 subunit A [Source:HGNC Symbol;Acc:HGNC:851] | 8013 | -0.156 | 0.0975 | No |
| 28 | ATP1A2 | ATPase Na+/K+ transporting subunit alpha 2 [Source:HGNC Symbol;Acc:HGNC:800] | 8218 | -0.173 | 0.0955 | No |
| 29 | ATP1A1 | ATPase Na+/K+ transporting subunit alpha 1 [Source:HGNC Symbol;Acc:HGNC:799] | 8282 | -0.177 | 0.1048 | No |
| 30 | ATP2A2 | ATPase sarcoplasmic/endoplasmic reticulum Ca2+ transporting 2 [Source:HGNC Symbol;Acc:HGNC:812] | 9015 | -0.233 | 0.0666 | No |
| 31 | ABCC9 | ATP binding cassette subfamily C member 9 [Source:HGNC Symbol;Acc:HGNC:60] | 9346 | -0.259 | 0.0618 | No |
| 32 | KCNJ8 | potassium inwardly rectifying channel subfamily J member 8 [Source:HGNC Symbol;Acc:HGNC:6269] | 9421 | -0.266 | 0.0774 | No |
| 33 | ATP13A1 | ATPase 13A1 [Source:HGNC Symbol;Acc:HGNC:24215] | 9939 | -0.315 | 0.0626 | No |
| 34 | ATP4A | ATPase H+/K+ transporting subunit alpha [Source:HGNC Symbol;Acc:HGNC:819] | 10417 | -0.363 | 0.0546 | No |
| 35 | ATP2C1 | ATPase secretory pathway Ca2+ transporting 1 [Source:HGNC Symbol;Acc:HGNC:13211] | 10432 | -0.364 | 0.0828 | No |
| 36 | ATP2C2 | ATPase secretory pathway Ca2+ transporting 2 [Source:HGNC Symbol;Acc:HGNC:29103] | 10580 | -0.382 | 0.1021 | No |
| 37 | ATP13A3 | ATPase 13A3 [Source:HGNC Symbol;Acc:HGNC:24113] | 11136 | -0.448 | 0.0949 | No |
| 38 | ATP2B1 | ATPase plasma membrane Ca2+ transporting 1 [Source:HGNC Symbol;Acc:HGNC:814] | 11648 | -0.524 | 0.0973 | No |
Table: GSEA details [plain text format]

  

Fig 2: GOMF\_ATPASE\_COUPLED\_CATION\_TRANSMEMBRANE\_TRANSPORTER\_ACTIVITY      
 Blue-Pink O' Gram in the Space of the Analyzed GeneSet

  

Fig 3: GOMF\_ATPASE\_COUPLED\_CATION\_TRANSMEMBRANE\_TRANSPORTER\_ACTIVITY: Random ES distribution      
 Gene set null distribution of ES for **GOMF\_ATPASE\_COUPLED\_CATION\_TRANSMEMBRANE\_TRANSPORTER\_ACTIVITY**
